# Effects of Composite Starter Cultures on Microbial Succession, Quality Characteristics and Flavor Profile of Air-Dried Mutton

**DOI:** 10.3390/foods15111964

**Published:** 2026-06-02

**Authors:** Jiaqi Zhang, Lina Sun, Erke Sun, Shiqi Hao, Hongbo Qu, Yanrong Chen, Weiqi Qin, Ye Jin, Lihua Zhao, Xueying Sun

**Affiliations:** 1 College of Food Science and Engineering, Inner Mongolia Agricultural University, Hohhot 010018, China; ybdgh957@163.com (J.Z.); beautysunlina@126.com (L.S.); sun15848107303@163.com (E.S.); haosq_imau@163.com (S.H.); quhongbo1214@163.com (H.Q.); cyrr5061@163.com (Y.C.); q15147561598@163.com (W.Q.); jinyeyc@sohu.com (Y.J.); 2 Integrative Research Base of Beef and Lamb Processing Technology, Inner Mongolia Agricultural University, Hohhot 010018, China

**Keywords:** traditional meat processing, physicochemical modification, fermentation optimization, microbial regulation

## Abstract

Air-dried mutton is a traditional, culturally significant meat product, yet its spontaneous fermentation is inherently constrained by unstable microbial communities, leading to batch-to-batch quality inconsistency and potential food safety hazards. Elucidating whether composite starter cultures can modulate the microbiota and enhance product quality is therefore critical for standardized industrial processing. Herein, we investigated the effects of a defined starter culture (composed of *Lactiplantibacillus plantarum* and *Pediococcus pentosaceus* in a 2:1 ratio, with a total inoculum of 10^8^ CFU/g) on the quality and flavor of air-dried mutton, comparing inoculated samples (FJ) with naturally fermented controls (ZR). The fermentation was conducted at 30 °C and 95% relative humidity (RH) for 24 h, followed by air-drying at 4 °C for 21 d, with all assays performed in three biological and three technical replicates. Starter inoculation significantly reduced the pH, water activity (Aw), total volatile basic nitrogen (TVB-N), and thiobarbituric acid reactive substances (TBARS) values while improving sensory acceptability (*p* < 0.05). Amplicon sequencing analysis revealed a lactic acid bacteria (LAB)-dominated microbiota in FJ samples, with elevated abundances of *Pediococcus* and *Lactobacillus* and reduced abundance of *Pseudomonas*. The inoculated group also exhibited altered eicosapentaenoic acid content and a more diverse volatile flavor profile, with eight key aroma compounds positively correlating with LAB abundance. These findings demonstrate that composite starter inoculation improves physicochemical quality, stabilizes the microbial community, and enhances flavor in air-dried mutton. Further mechanistic validation and scale-up trials are required to confirm industrial applicability.

## 1. Introduction

Air-dried mutton is a traditional meat product produced from fresh mutton through a series of processes, including curing, sun-drying, and air-drying. Traditional air-dried meat products are widely distributed in China, with the highest concentrations in the Qinghai–Tibet Plateau, Xinjiang, Inner Mongolia, and other regions [1]. Air-dried mutton is popular among consumers for its compact size, high nutritional content, and long shelf life, occupying an important position in the meat market [2] and boosting the industrial development of traditional regional air-dried meat products in China. However, restricted by traditional processing modes and evolving market demands, this product is plagued by problems such as non-standardized technology, uneven quality, and poor batch consistency, while defects like coarse texture, dull color, and monotonous flavor are becoming increasingly prominent [3]. Spontaneous fermentation results in uncontrollable microbial succession and increases the risk of quality deterioration [4]. Notably, existing research on air-dried mutton mainly focuses on the investigation of natural microbial communities and conventional quality detection. The targeted regulation of microbial succession by exogenous composite starters, the internal metabolic mechanisms linking microbial flora to flavor formation, and the differentiation of metabolic pathways between single-strain and mixed-strain fermentation remain poorly understood and insufficiently clarified. Thus, optimizing processing technology to realize standardized, industrialized, and safe production is the key direction for the high-quality development of this industry.

Lactic acid bacteria (LAB) commonly used in food fermentation include *Lactobacillus*, *Bifidobacterium*, *Pediococcus*, and other genera [5]. In meat products, LAB play a multifaceted role. They produce organic acids to lower the pH, generate antimicrobial substances to inhibit the growth of spoilage and pathogenic bacteria, improve textural properties, stabilize color, and promote the breakdown of proteins and lipids to yield flavor precursors, including free fatty acids and amino acids, ultimately enhancing both the nutritional value and flavor quality of the final products [6]. Unlike individual strains, composite starter cultures can exert synergistic metabolic effects on multiple functionally complementary strains. *Lactiplantibacillus plantarum* has excellent acid-producing and antibacterial properties, which can rapidly reduce the pH of the system and inhibit spoilage bacteria, while *Pediococcus pentosaceus* has strong enzyme-producing and flavor-producing capabilities, which can promote the decomposition of proteins and lipids and thus generate flavor precursors [7]. This functional complementarity enables composite starters to achieve better performance in flavor optimization, color regulation, and sensory quality improvement than single strains while also improving the batch stability of products. Liu et al. [8] found that dry-fermented sausages inoculated with lactic acid bacterium strains *Lactobacillus delbrueckii* N102 and *Latilactobacillus sakei* H1-5 ensured product safety through rapid acidification and inhibition of the spoilage bacterium *Enterobacter putrefaciens* and contributed to the formation of flavor precursors. Mani-López et al. [9] found that salami-type fermented meat products effectively reduced the pH of the final product via LAB-driven acidification, establishing a physicochemical environment unfavorable for the growth of spoilage microorganisms. Their study further confirmed the impacts of LAB strains on product quality and safety. Starter cultures are widely used in the field of meat product production. Again, unlike individual strains, composite starter cultures can exert synergistic metabolic effects on multiple strains and offer more significant advantages in terms of flavor optimization, color regulation, and sensory quality improvement [10]. Zheng et al. [11] found that fermented sausages inoculated with mixed starter cultures exhibited higher redness values and overall sensory acceptability in comparison with those inoculated with single-strain starter cultures.

Herein, we put forward our core hypothesis: composite starter inoculation can reshape the microbial succession of air-dried mutton, regulate lipid and protein metabolic pathways, inhibit the proliferation of spoilage microbes, and ultimately improve physicochemical quality and flavor characteristics. The introduction of exogenous starter cultures remodels the bacterial community structure during air-dried mutton processing. Microbial succession represents a core intrinsic factor determining meat quality [12]. In this study, we aimed to clarify the effects of composite starter cultures on the microbial succession and quality development of air-dried mutton and reveal the correlation between microbial community dynamics and flavor-metabolic characteristics. The study findings will provide theoretical support for standardized industrial production of traditional air-dried mutton and the traditional practice of preparing ethnic meat products.

## 2. Materials and Methods

### 2.1. Test Materials and Sample Preparation

Fresh sheep hind leg meat from 6–8-month-old castrated male Small-tailed Han sheep, raised under standardized indoor feeding conditions by Inner Mongolia Juyang Animal Husbandry Co., Ltd., Hohhot, China, exhibited initial physicochemical properties: pH 5.72 ± 0.03, moisture 75.23 ± 0.45%, crude protein 20.15 ± 0.32% and crude fat 3.68 ± 0.21%, and initial microbial counts: total viable count 3.2 × 10^4^ CFU/g and lactic acid bacteria count 1.5 × 10^3^ CFU/g. The meat was transported under cold-chain conditions at 4 °C, stored at 4 °C, trimmed to remove fascia, excess fat and connective tissue, cut into uniform strips of 5 cm × 3 cm × 2 cm, and cured with 3% (*w*/*w*) sodium chloride for 12 h at 4 °C.

*Lactiplantibacillus plantarum* and *Pediococcus pentosaceus*, provided by the innovation team’s laboratory [13] and confirmed to have excellent acid-producing and antibacterial properties as well as good enzyme-producing and flavor-producing capacity consistent with those of functional strains commonly used in fermented meat products, were used to establish two groups: a natural air-dried control group (ZR) and a composite starter-inoculated test group (FJ). The FJ group was uniformly inoculated with 10^8^ CFU/g composite starter (*L. plantarum: P. pentosaceus* = 2:1) via surface spraying and gentle rubbing, then fermented for 24 h at 30 °C and 95% RH in a calibrated LHS-10CL constant-temperature and -humidity incubator, with real-time monitored temperature and RH fluctuating within ±0.5 °C and ±2% RH. The ZR group received no starter inoculation and directly entered the air-drying stage after curing. Three independent production batches served as biological replicates, with three parallel samples per batch as technical replicates and a single 500 g mutton block as the experimental unit. All samples were air-dried for 21 d at 4 °C and 51% RH in a closed, ventilated constant-temperature and -humidity chamber, with continuous temperature and RH monitoring and an axial-flow circulation system ensuring uniform drying.

### 2.2. Test Reagents

Analytical-grade reagents, including trichloroacetic acid, sodium chloride, magnesium oxide, boric acid, thiobarbituric acid, n-hexane, chloroform, anhydrous sodium sulfate, sodium hydroxide, sulfuric acid, anhydrous ethanol, and sodium bicarbonate, were purchased from Sinopharm Chemical Reagent Co., Ltd., Shanghai, China. Chromatographic-grade methanol and acetonitrile were obtained from Thermo Fisher Scientific Inc., Waltham, MA, USA, while analytical-grade acetone was supplied by Tianjin Fengchuan Chemical Reagent Technology Co., Ltd., Tianjin, China.

### 2.3. Instruments and Equipment

An HD-3A intelligent water activity meter was purchased from Wuxi Huake Instrument and Meter Co., Ltd., Wuxi, China. An AL204 1/10,000 electronic analytical balance and a PB-STAR direct carcass pH meter were obtained from Mettler-Toledo Instruments Co., Ltd., Shanghai, China. A TCP2 automatic color difference meter was supplied by Beijing Aoyikemi Optoelectronics Co., Ltd., Shanghai, China. A Trace 1300 ISQ gas chromatography–mass spectrometry (GC-MS) system and 1260 high-performance liquid chromatography (HPLC) system were purchased from Thermo Fisher Scientific, Waltham, MA, USA. Additionally, an HH-2 digital display constant-temperature water bath was provided by Jintan Jiangnan Instrument Factory, Jintan, China, and an LHS-10CL constant-temperature and -humidity chamber was purchased from Shanghai Yiheng Technology Co., Ltd., Shanghai, China.

### 2.4. Test Methods

#### 2.4.1. Methods of Determining Physicochemical Quality

Triplicate parallel tests were established for all indices, and the results were averaged. The following determination methods were used.

##### pH Value

The pH values of the meat samples were determined with reference to the method reported by Lin et al. [14], with minor adjustments. Specifically, 2 g of a meat sample was precisely weighed and homogenized with 18 mL of sterile normal saline. The mixture was magnetically stirred for 30 min to obtain a homogenate, and the pH value was then directly measured using a pH direct meter.

##### Aw

Aw was measured according to Li et al.’s method [15] with minor modifications. Briefly, 10 g of chopped meat samples was weighed and spread evenly in a dedicated measuring cup. After sealing and complete equilibration, Aw was determined using a water activity meter.

##### Moisture Content

Moisture content: Moisture content was determined via the direct drying method as per Ren et al.’s approach [16] with minor modifications. Briefly, fresh chopped meat samples were accurately weighed and spread uniformly in pre-dried aluminum crucibles. All samples were placed in a drying oven at 105 °C and heated to a constant weight. The moisture content was calculated according to the variation in weight before and after drying.

(1) $$\mathrm{Moisture}\;\mathrm{content}\;(\%)\;=\frac{m_{1}-m_{2}}{m_{1}-m_{0}}\times 100$$

Symbol definition

*m*_0_ = mass of empty aluminium dish (g)

*m*_1_ = mass of dish + sample before drying (g)

*m*_2_ = mass of dish + sample after drying to constant weight (g)

##### Color Difference

The lightness value (L*), redness value (a*), and yellowness value (b*) of samples were determined using a TCP2 automatic color difference meter, following the method described by Bai et al. [17] with minor modifications. The colorimeter was calibrated with a standard white ceramic plate (L* = 94.52, a* = −0.78, b* = 1.67) before measurement. Measurements were performed using a D65 standard illuminant and 10° observer angle. For each sample, three different flat muscle surfaces (avoiding visible fat and fascia) were measured, and the average value was calculated.

##### TVB-N

Lean meat with the fascia removed was minced and homogenized. Subsequently, 10 g of the sample was accurately weighed, added to 100 mL of distilled water, oscillated for 30 min, and left to stand for 10 min. Then, 25 mL of the supernatant was absorbed into a distillation tube, 1 g of magnesium oxide was added, and distillation determination was carried out with 30 mL of boric acid absorbing solution and a distillation time set to 180 s, following the method described by Kim et al. [18] with minor modifications.

##### TBARS

Lean meat with the fascia removed was minced and mixed evenly. Then, 5 g of the sample was accurately weighed, added to 25 mL of trichloroacetic acid solution, oscillated for mixing, and filtered. The filtrate was mixed with TBARS chromogenic reagent and then reacted in a constant-temperature water bath, cooled, and treated with trichloromethane. Absorbance was measured at wavelengths of 532 nm and 600 nm, with a blank control set simultaneously, as per the method described by Li et al. [19] with minor modifications.

#### 2.4.2. Determination of Fatty Acid Content

Lipid extraction and sample pretreatment for fatty acid determination were performed with reference to the method established by Lucarini et al. [20] with minor modifications, and the details of the procedures are as follows: Briefly, a 10 g minced-meat sample was accurately weighed, mixed with 100 mL of a chloroform–methanol mixture (with a volume ratio of 2:1), and subjected to oscillatory extraction at 55 °C for 30 min. The filtrate obtained was concentrated via rotary evaporation to recover the crude fat fraction. The resulting crude fat was saponified in 1 mol/L NaOH solution in a 75 °C water bath for 2 h, and then acidification with 1 mol/L H_2_SO_4_ was carried out. Subsequently, 50 mL of n-hexane was added to the acidified system, and micro-boiling distillation extraction was carried out for 2 h. The organic extract was collected, dehydrated over anhydrous Na_2_SO_4_, concentrated to 1 mL via rotary evaporation, and finally adjusted to a constant volume of 2 mL with n-hexane for subsequent determination.

#### 2.4.3. Sensory Evaluation

Sensory quality assessment of air-dried mutton samples was conducted using a categorical scoring method, with reference to the procedure reported by Wang et al. [21], with minor modifications. An evaluation panel consisting of 10 assessors with a professional food science background (male-to-female ratio of 1:1, aged 22–35 years, no olfactory/gustatory disorders, non-smokers) was recruited for the test. All panelists received 8 h of systematic standardized training covering air-dried mutton sensory characteristics, scoring criteria explanation, and reference sample calibration to unify evaluation standards. A pre-experiment confirmed excellent inter-panelist consistency with a Kendall’s coefficient of concordance (W) of 0.87 (*p* < 0.01).

Prior to evaluation, all samples were equilibrated to room temperature (22 ± 2 °C) for 30 min, trimmed to remove surface impurities, and cut into uniform 2 mm thick slices. Consistent with the traditional consumption habit of Inner Mongolian air-dried mutton, samples were evaluated directly in their raw state without additional cooking. Samples were randomly coded with three-digit numbers and presented in a random order to eliminate order bias. After tasting each sample, panelists immediately rinsed their mouths with purified water, and a 30 s rest interval was set before the evaluation of the next sample to prevent carryover effects. Each sample was evaluated independently by all panelists on four dimensions (Color Attributes, Flavor Characteristics, Morphological Structure, Taste Profile), as detailed in Table 1. Finally, the scores of all individual indicators were aggregated to calculate the total sensory score. All evaluations were repeated three times on separate days to verify reproducibility, ensuring the reliability of the sensory data.

### 2.5. Determination of Microbial Community Structure

Total microbial community deoxyribonucleic acid (DNA) extraction and polymerase Chain Reaction (PCR) amplification pretreatment were performed with reference to the method described by Lian et al. [22] with minor modifications. The detailed procedures are as follows: Total microbial community DNA was extracted from the samples, for which the instructions included with the E.Z.N.A.^®^ Soil DNA Extraction Kit (Omega Bio-tek, Norcross, GA, USA)were strictly followed. The integrity of the extracted DNA was verified via 1% agarose gel electrophoresis, while the concentration and purity of the DNA were quantified using a NanoDrop 2000 Ultramicro Spectrophotometer (Thermo Fisher Scientific, Waltham, MA, USA Software version: 1.6). PCR amplification was subsequently carried out using barcode-tagged specific primers, with the qualified genomic DNA serving as the template. For full-length amplification of the 16S rRNA gene, the primer pair 27F (5′-AGRGTTYGATYMTGGCTCAG-3′) and 1492R (5′-RGYTACCTTGTTACGACTT-3′) was employed as described in [23], while primers ITS1F and ITS4R were used for full-length amplification of the Internal Transcribed Spacer (ITS) region according to [24].

The total volume of the PCR reaction system was 20 μL, and the components were as follows: 4 μL of 5× FastPfu Buffer, 2 μL of 2.5 mmol/L dNTPs, 0.8 μL each of 5 μmol/L forward and reverse primers, 0.4 μL of FastPfu DNA Polymerase (TransGen Biotech Co., Ltd., Beijing, China), 0.2 μL of Bovine Serum Albumin (BSA, Sigma-Aldrich, St. Louis, MO, USA), and 10 ng of template DNA, and the remaining volume was made up with sterile ddH_2_O. Triplicate replicates were set for each sample.

The PCR amplification procedure was as follows: pre-denaturation at 95 °C for 3 min; 27 cycles of denaturation at 95 °C for 30 s, annealing at 60 °C for 30 s, and extension at 72 °C for 30 s; a final extension at 72 °C for 10 min; and storage of the amplicons at 4 °C. After verification via 2% agarose gel electrophoresis, the amplicons were purified using magnetic beads, and the concentration was quantified with a Qubit 4.0 Fluorometer. The purified products were pooled in equal proportions according to the sequencing depth requirements.

The sequencing library was constructed using the SMRTbell Prep Kit 3.0 kit, with the core workflow including DNA damage repair, end repair, and adapter ligation. Shanghai Majorbio Bio-pharm Technology Co., Ltd. (Shanghai, China) was commissioned to perform the sequencing work, and full-length sequencing was performed using the Pacbio Sequel IIe sequencing platform. The raw off-machine data were processed using SMRT-Link v 11.0 software in Circular Consensus Sequencing (CCS) mode to generate high-fidelity (HiFi) reads for subsequent bioinformatics analysis.

### 2.6. Determination of Volatile Flavor Compounds

Volatile flavor compounds in the samples were determined via solid-phase microextraction–gas chromatography–mass spectrometry (SPME-GC-MS) with reference to the method described by Jiang et al. [25] with minor modifications. The details regarding the procedures and instrumental parameters employed are as follows: A 5 g minced sample was accurately weighed and sealed in a 20 mL headspace vial. After incubation in a water bath at 60 °C, volatile compounds were adsorbed by headspace solid-phase microextraction fibers for 40 min, desorbed at 250 °C for 3 min, and then injected for instrumental analysis. The GC conditions were as follows: the capillary column measured 30 m × 0.25 mm × 0.25 μm; the carrier gas was He with a flow rate of 1.0 mL/min; the transfer line temperature was 250 °C; splitless injection was applied for 1 min; and the temperature program consisted of holding an initial temperature of 40 °C for 3 min, then ramping it to 150 °C at 4 °C/min and holding it for 1 min, then ramping it to 200 °C at 5 °C/min, and finally ramping it to 230 °C at 20 °C/min and holding it for 5 min. The MS conditions were as follows: ion source temperature, 250 °C; mass scan range, 30–400 *m*/*z*; and solvent delay, 1.0 min. Qualitative and quantitative analysis: Mass-spectrum retrieval was performed against the MEANLIB, NIST Demo, and Wiley Library mass-spectral databases, with a matching degree > 800 set as the valid qualitative criterion. The relative content of each component was expressed as the percentage of its peak area relative to the total peak area of all identified compounds.

The contribution of each aroma component to the overall flavor of the sample is usually expressed using the Relative Odor Activity Value (ROAV).

The formula for calculating the ROAV is as follows:

(2) ROAV=100×CA÷CMAX × TMAX÷TA

Here, CA is the relative percentage content of a specific volatile flavor compound A, CMAX is the relative percentage content of the volatile flavor compound with the greatest contribution to the overall flavor of the sample, TMAX is the odor threshold of the volatile flavor compound with the greatest contribution to the overall flavor of the sample, and TA is the odor threshold of the specific volatile flavor compound A.

### 2.7. Statistical Analysis of Data

All bioinformatics analyses of microbial communities were performed using the Majorbio Cloud Platform [26]. The alpha diversity indices of microbial communities were calculated with Mothur (version 1.48.0; Schloss Lab, University of Michigan, Ann Arbor, MI, USA); given the non-normal distribution of the data, intergroup comparisons of alpha diversity were carried out via the non-parametric Kruskal–Wallis H test. LEfSe analysis (LDA score > 2, *p* < 0.05) was then applied to identify microbial taxa with significantly different abundances across sample groups [27]. Statistical analyses of physicochemical indices, sensory evaluation results, and volatile flavor compounds were conducted in SPSS 26.0. Prior to parametric testing, the Shapiro–Wilk test and Levene’s test were applied to assess data normality and homogeneity of variances, respectively. Data meeting the criteria of normality and homogeneous variances were subjected to one-way analysis of variance (ANOVA), with post hoc pairwise comparisons performed using Duncan’s multiple range test. Non-normally distributed data, by contrast, were analyzed via the Kruskal–Wallis H non-parametric test for intergroup comparisons. All statistical analyses were performed at a significance level of *p* < 0.05, and the results are presented as means ± standard deviations (SDs). Pearson correlation analysis was conducted to explore relationships between dominant bacterial genera (relative abundance > 1%), physicochemical indices, and key flavor compounds, with corresponding correlation network diagrams generated to visualize these associations. All final data visualizations were created using Origin 2018 software (OriginLab Corporation, Northampton, MA, USA; Version 9.5.1.195).

## 3. Results

### 3.1. Physicochemical Quality of Fermented Air-Dried Mutton

Compared with the naturally air-dried control group (ZR), the core quality indices of the test group (FJ) inoculated with the composite starter were significantly better (*p* < 0.05). As shown in Table 2, FJ had a pH of 5.40, an Aw of 0.60, a moisture content of 6.01%, a TVB-N content of 5.36 mg/100 g, and a TBARS value of 0.34 mg/100 g, all of which were significantly lower than those of ZR (*p* < 0.05). The instrumental redness value (a*) of the FJ group was 13.51, the sensory color attributes score was 21.50, and the total sensory score was 84.21, all of which were significantly higher than those of the ZR group (*p* < 0.05).

The significant differences in the above indices may primarily derive from the metabolic activity of LAB in the composite starter. These bacteria’s acid-producing property can rapidly reduce the pH of the system and create a synergistic antibacterial environment with low Aw, thus reducing protein spoilage and lipid oxidation, an effect similar to the common regulation pattern of LAB in fermented meat products.

### 3.2. Microbial Community Structure of Fermented Air-Dried Mutton

**Figure 1:**
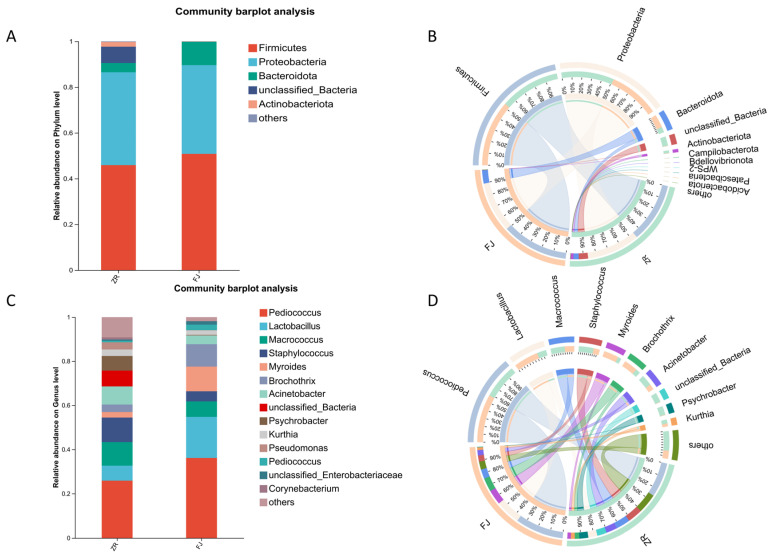
Bacterial community composition. (**A**) Diagram of the composition of the bacterial community in fermented air-dried meat at the genus level. (**B**) Diagram of the composition of the bacterial community in fermented air-dried meat samples at the phylum level. (**C**) Circos plot of the bacterial community at the phylum level in fermented air-fried meat. (**D**) Circos plot of the bacterial community at the genus level in fermented air-dried meat.

As shown in Table 3, high-throughput sequencing of the bacterial communities in the two groups yielded 99,927 valid sequences with a length of 421–429 bp. The number of amplicon sequence variants (ASVs) in FJ was 118, which was significantly lower than the value of 263 in ZR (*p* < 0.05). The Ace, Chao, and Shannon indices for ZR were significantly higher than those for FJ (*p* < 0.05). As presented in Figure 1A,B, at the phylum level, Firmicutes and Proteobacteria were the dominant phyla in both groups, and the relative abundance of Firmicutes in FJ was significantly higher than that in ZR (*p* < 0.05). As presented in Figure 1C,D, at the genus level, *Pediococcus* and *Lactobacillus* were the absolute dominant genera in FJ, with extremely low abundance of *Pseudomonas*; the abundances of *Psychrobacter* and *Brochothrix* in ZR were significantly higher than those in FJ (*p* < 0.05).

The distinct microbial community structure between groups was possibly associated with the selective pressure of the complex starter culture and the subsequent adjustment of the ecological niche, a situation that is similar to the succession difference between spontaneous fermentation and inoculated fermentation. At the phylum level, the absolute dominant status of Firmicutes in FJ matches was consistent with the core role of this phylum in LAB fermentation, as most functional starter strains for fermented meat products belong to Firmicutes. At the genus level, the lower relative abundance of spoilage-related bacteria such as *Pseudomonas* and *Psychrobacter* in FJ was consistent with the lower TVB-N and TBARS values in the physicochemical results, which suggests a potential association with the composite starter’s ability to improve safety.

### 3.3. Microbial Community Functional Prediction of Fermented Air-Dried Mutton

**Figure 2:**
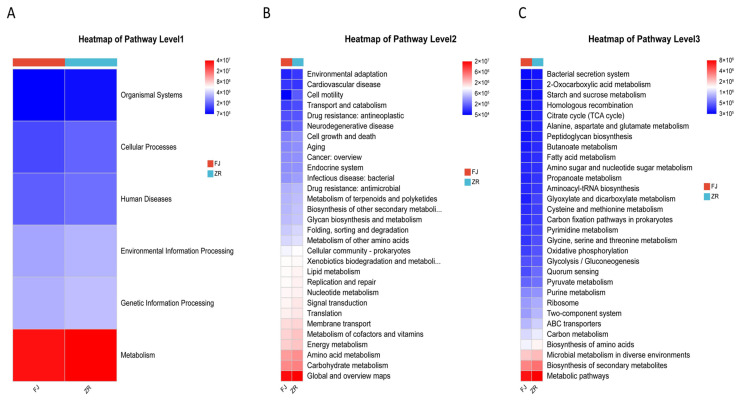
Functional prediction of microbial communities in fermented air-dried meat. (**A**) Heatmap of Kyoto Encyclopedia of Genes and Genomes (KEGG) pathway at Level 1 in fermented air-dried meat samples. (**B**) Heatmap of KEGG pathway at Level 2 in fermented air-dried meat samples. (**C**) Heatmap of KEGG pathway at Level 3 in fermented air-dried meat samples.

To further explore the functional differences of microbial communities between the two groups, PICRUSt2 analysis was performed based on the 16S rRNA gene sequencing data and the Kyoto Encyclopedia of Genes and Genomes (KEGG) database (Figure 2). At Level 1, Metabolism was the dominant pathway in both groups, with slightly higher abundance in the FJ group than in the ZR group. At Level 2, FJ was enriched in core metabolic pathways (carbohydrate, amino acid, and energy metabolism), while ZR showed relatively higher abundance of disease-related pathways. At Level 3, FJ exhibited specific enrichment of flavor-relevant pathways, including Metabolic pathways, Biosynthesis of secondary metabolites, and amino acid biosynthesis. These results indicate that composite starter inoculation reshaped the microbial functional profiles toward enhanced metabolic activity in the FJ group. These results indicate that composite starter inoculation reshaped the microbial functional profiles, directing the microbiota toward enhanced metabolic activity beneficial for product quality.

### 3.4. Free Fatty Acid Profile of Fermented Air-Dried Mutton

As can be seen from Table 4, a total of 27 fatty acids were detected in both groups, including 12 saturated fatty acids (SFAs), 6 monounsaturated fatty acids (MUFAs), and 7 polyunsaturated fatty acids (PUFAs). The contents of lauric acid and cis-8,11,14-eicosatrienoic acid in the FJ group were significantly higher than those in the ZR group (*p* < 0.05), and EPA was detected only in the FJ group. Notably, relatively large differences in γ-linolenic acid and palmitic acid were observed between the two groups, which may be closely associated with the distinct microbial composition and metabolic characteristics. All fatty acid measurements were performed with three biological replicates and consistent results were obtained, supporting the reliability of the data. The contents of oleic acid, palmitoleic acid, γ-linolenic acid, and docosadienoic acid (DDA) in the ZR group were significantly higher than those in the FJ group (*p* < 0.05).

The significant difference in fatty acid composition between the two groups may be associated with the differentiation of fat-metabolizing capacities of different microbial communities. Different genera may differ in the expression levels and substrate preferences of extracellular lipases: the dominant *Lactobacillus* and *Pediococcus* in the FJ group may be related to the release and enrichment of lauric acid and functional polyunsaturated fatty acids, while miscellaneous bacteria represented by *Psychrobacter* in the ZR group may be associated with the higher levels of monounsaturated fatty acids such as oleic acid and palmitoleic acid, which may partly explain the significant differentiation of fatty acid profiles between the two groups. Meanwhile, the low pH environment associated with LAB fermentation may influence the activity of endogenous lipases in mutton, further contributing to the difference in fatty acid composition, laying a material foundation for the subsequent differentiation of flavor substances as the core precursors of flavor formation.

### 3.5. Volatile Flavor Compounds of Fermented Air-Dried Mutton

**Figure 3:**
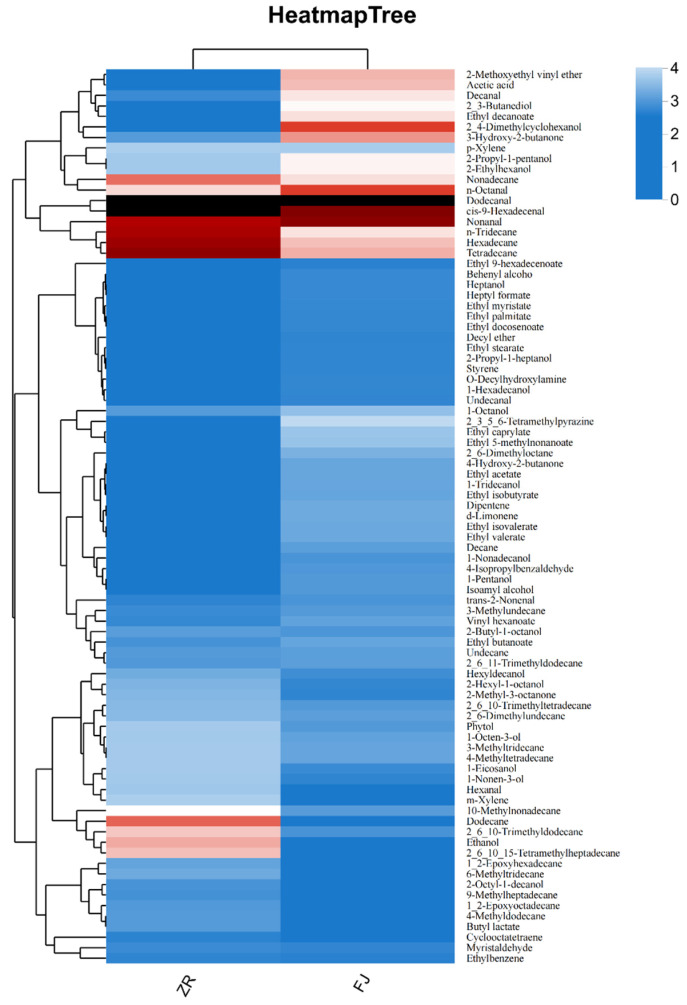
Clustering heat map of volatile flavor compounds in fermented air-dried. Note: The color gradient from blue to red indicates the relative abundance of volatile flavor compounds, with red representing high abundance and blue representing low abundance. The black dendrograms on the left and top represent the results of hierarchical clustering analysis, showing the similarity of volatile flavor profiles between different samples and among different compounds.

A total of 72 volatile flavor compounds were detected in the FJ group, while only 48 were found in the ZR group, showing a significant difference in flavor composition between the two groups (*p* < 0.05), as presented in Figure 3. Using ROAV > 1 as the criterion, eight key flavor compounds were screened: vinyl hexanoate, ethyl acetate, ethyl decanoate, ethyl octanoate, nonanal, decanal, 2,3-butanediol, and 2,3,5,6-tetramethylpyrazine. Six flavor-modifying compounds with 0.1 ≤ ROAV ≤ 1 were also identified, with distinct compositions between the two groups. Odor threshold data were cited from the literature and collated, as shown in Table 5 [28,29].

The significant differences in the types and compositions of flavor compounds between the two groups may be associated with dual variations in fatty acid metabolism and microbial metabolic activity. The eight key flavor compounds screened in this study are all closely related to fatty acid oxidative degradation and LAB metabolism: aldehydes are mainly derived from the directional oxidation of unsaturated fatty acids, while esters are generally generated by the esterification of alcohol precursors from LAB metabolism and free fatty acids. The richer flavor compounds in the FJ group may be related to its stable functional flora, which promotes the directional generation of pleasant flavor substances while reducing the formation of off-flavor compounds from non-specific fatty acid oxidation. The unique characteristic metabolites of LAB, such as 2,3-butanediol and 2,3,5,6-tetramethylpyrazine, exclusively detected in the FJ group, further suggest that starter inoculation contributes to the flavor complexity of the product, which is consistent with the classic mechanism of flavor formation in meat products.

### 3.6. Analysis of the Correlation Between Bacterial Community and Quality Indices

**Figure 4:**
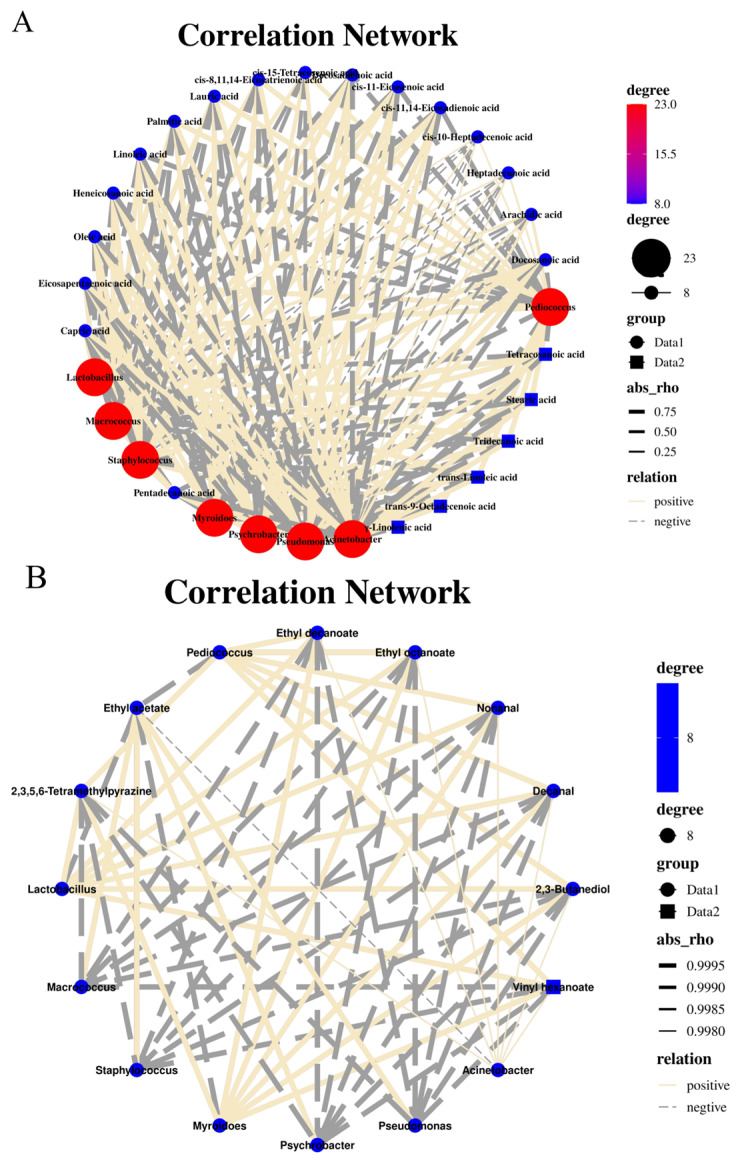
Correlation plot. (**A**) Correlation analysis plot of microbial community structure and flavor compounds in fermented air-dried mutton. (**B**) Correlation analysis plot of microbial community structure and fatty acid content in fermented air-dried mutton.

Pearson correlation and Spearman rank correlation were used to analyze the relationships between dominant genera (relative abundance > 1%) and free fatty acids as well as volatile flavor compounds. As shown in Figure 4, the dominant genera were clearly clustered into two groups: the group dominated by *Pediococcus* and *Lactobacillus* showed extremely significant positive correlations with most functional fatty acids and key flavor compounds (*p* < 0.01), whereas the group dominated by *Staphylococcus* and *Psychrobacter* exhibited the opposite correlation trend.

This completely opposite correlation trend indicates that the two groups of bacteria may play distinct roles in the quality development of air-dried mutton, and the LAB-dominated group is closely linked to the optimization of product quality. Specifically, the significant positive correlations of *Pediococcus* and *Lactobacillus* with functional fatty acids and key flavor compounds further suggest the potential involvement of these two dominant genera in fatty acid metabolism and flavor formation, which is consistent with the findings from physicochemical and microbial community analyses. In contrast, the negative correlations of Staphylococcus and *Psychrobacter* with high-quality indicators imply that these genera may not contribute to quality improvement and may even be associated with quality deterioration to a certain extent. These clustering and correlation results also provide potential target references for improving air-dried mutton quality via microbial regulation.

## 4. Discussion

Traditional air-dried mutton is a distinctive regional meat product, yet the industrial advancement of the production of this product is severely restricted by uncontrollable spontaneous fermentation and inconsistent batch-to-batch quality. The fermentation stage is the core driver of quality formation, as microbial succession during this period directly determines subsequent stability and final quality. Accordingly, this work explores the regulatory effects of a composite starter culture composed of *Lactiplantibacillus plantarum* and *Pediococcus pentosaceus* during fermentation and its subsequent effects on the quality, microbial succession, and flavor development of air-dried mutton.

As a traditional fermented meat representative of pastoral areas of Inner Mongolia, air-dried mutton has profound local dietary characteristics and greatly supports the sustainable development of the regional mutton industry. Unlike standardized fermented meat commodities such as European dry-cured ham and Chinese Cantonese sausage, traditional air-dried mutton is generally manufactured in natural open environments without precise temperature and humidity control, with fermentation and maturation relying entirely on indigenous microflora from raw meat and processing surroundings and no targeted regulation of the core fermentation stage. While this natural processing route preserves the product’s unique regional flavor profiles, it inevitably results in an unstable microbial composition during fermentation, leading to significant batch-to-batch heterogeneity and hindering large-scale industrial promotion. Current studies focus on single-strain effects or end-point quality, while composite starter-mediated microbial regulation and its systematic links to quality remain underexplored.

Composite starter supplementation during fermentation may be associated with improved physicochemical quality and edible safety of air-dried mutton through inter-strain functional complementarity. *L. plantarum-driven* acidification and *P. pentosaceus*-mediated color stabilization exert synergistic effects, consistent with LAB regulatory mechanisms in fermented meat systems [30,31]. *L. plantarum* may contribute to the reduction of matrix pH and water activity (Aw) during fermentation, thereby helping to construct a dual antibacterial barrier at the early processing stage that may suppress spoilage microbial growth and reduce TVB-N and TBARS accumulation throughout the subsequent air-drying process. Meanwhile, *P. pentosaceus* may contribute to meat redness through its potential reducing capacity during fermentation, which aligns with the reported color-stabilizing properties of LAB in cured and fermented meat [32,33]. Furthermore, mixed starter inoculation may influence muscle protein denaturation during fermentation, thus contributing to the final textural properties of air-dried products. Moderate and rapid pH reduction in the fermentation stage may help inhibit undesirable microbes in early processing, while maintaining favorable texture and color stability during long-term air-drying. Traditional Inner Mongolian air-dried mutton is typically circulated and stored at ambient temperature without cold chain support. Thus, the combined reduction in pH and Aw established during fermentation is particularly critical for inhibiting pathogenic bacteria, including Listeria monocytogenes and Salmonella, throughout processing and long-term storage. These effects reflect the strain-specific functional complementarity of the composite starter, which is superior to single-strain fermentation in comprehensive quality improvement [10,11].

Composite starter inoculation was associated with marked changes in the microbial community structure of air-dried mutton and may influence the subsequent succession of microflora throughout the air-drying process. The starter rapidly colonizes and dominates the microbiota via niche competition, consistent with inoculation effects in fermented meats [34,35]. The fermentation stage is a key period for the formation and stabilization of microbial community structure in air-dried mutton: the composite starter may achieve rapid colonization and proliferation during fermentation, reasonably decrease microbial diversity, enable *Lactobacillus* and *Pediococcus* to rapidly dominate the fermentation system at the early processing stage, and be associated with the lower relative abundance of typical spoilage microorganisms such as *Pseudomonas*. The elevated abundance of Firmicutes in the final product was possibly related to the proliferation of the two inoculated LAB strains during fermentation, both of which belong to this phylum [36]. The suppression of spoilage taxa established in the fermentation phase further strengthened microbial safety throughout the entire processing [37]. High-throughput sequencing results clearly distinguished community differences between natural spontaneous fermentation and artificial inoculation-regulated fermentation. In contrast, artificial inoculation enabled functional LAB strains to occupy dominant ecological niches during fermentation, establishing a stable fermentation microecosystem that remained consistent throughout air-drying. Dominant LAB populations continuously produced organic acids and antibacterial metabolites to restrict spoilage growth during processing, and their predictable metabolic activities enabled directional regulation of the entire production process, providing a theoretical basis for standardized industrial production. Functional prediction results further supported the regulatory role of composite starters on microbial metabolism. The enhanced enrichment of carbohydrate, amino acid and energy metabolism pathways in the FJ group is consistent with the higher lipolytic and proteolytic activities of LAB, which provide sufficient precursors for flavor formation. In contrast, the higher abundance of disease-related pathways in the ZR group reflects the potential safety risks associated with spontaneous fermentation, which aligns with the higher TVB-N and TBARS values observed in this group. These functional differences provide a molecular-level explanation for the quality disparities between the two groups.

Correlation analysis demonstrated that LAB-dominated microbiota established during fermentation served as the core driver regulating fatty acid metabolism and flavor evolution in air-dried mutton [38]. LAB-secreted lipases and proteases drive precursor formation, consistent with microbial metabolic roles in fermented meats. The fermentation stage is a key period for flavor precursor formation and the initiation of fatty acid metabolism: the composite starter secreted lipase and protease during fermentation, decomposing muscle protein and fat to generate free amino acids and free fatty acids, which laid the critical material foundation for characteristic flavor formation via Maillard reaction and lipid oxidative decomposition in the subsequent air-drying process. Composite starter supplementation during fermentation was associated with changes in multiple beneficial functional fatty acids, including EPA, which exerts positive physiological benefits for human cardiovascular health [39]. Differences in fatty acid profiles between groups may be related to the distinct microbial community structure formed during fermentation [40]. In addition, mixed LAB fermentation increased the abundance and diversity of volatile compounds, especially esters, which are widely recognized as key aroma contributors in fermented meat [41]. Starter metabolism during fermentation also restrained lipoxygenase activity and reduced oxidative volatile by-products, ultimately improving the overall flavor performance of the final product [42,43]. Fatty acid profiling confirmed that targeted fermentation regulation via composite starters not only enhanced product safety but also upgraded its nutritional value. The large differences in γ-linolenic acid and palmitic acid between groups further reflect the distinct fatty acid metabolic patterns shaped by different microbial communities. The eight key aroma-active compounds screened via ROAV analysis jointly constructed the characteristic flavor profile of high-quality fermented mutton. Among them, esters such as ethyl acetate and ethyl decanoate contributed pleasant fruity and milky notes, effectively masking undesirable mutton off-flavors; 2,3-butanediol provided a mild, sweet taste, while 2,3,5,6-tetramethylpyrazine endowed a unique nutty and roasted aroma, further enriching the flavor hierarchy. Correlation results validated the central regulatory role of dominant LAB established during fermentation in metabolic modulation. Significant positive correlations between the two dominant genera, functional fatty acids, and key flavor compounds provide a clear targeted strategy for directional quality and flavor improvement of air-dried mutton via precise regulation of the fermentation stage.

The core innovation of this study is that it provides descriptive and correlative evidence for the important role of the fermentation stage in the quality formation of traditional Inner Mongolian air-dried mutton, suggests the potential effects of composite starter-mediated fermentation on product quality, and constructs a preliminary relational framework linking starter inoculation, microbial succession, fatty acid composition, and flavor formation. The fermentation strategy established in this study features simple operation, low cost, and no need for large-scale equipment modification, indicating potential applicability to the actual production conditions of small and medium-sized meat enterprises in Inner Mongolia. This research offers preliminary theoretical support and a technical reference for the standardized production and quality upgrading of traditional air-dried mutton by targeting the fermentation stage. Nevertheless, this study has certain limitations: the absence of single-strain control groups limits the in-depth elucidation of the individual contributions of each strain and their synergistic mechanisms, and large-scale industrial verification of the fermentation strategy remains lacking. Therefore, future research will focus on industrial scale-up trials of the fermentation process, optimization of process parameters for large-scale production, reduction in starter application costs, and in-depth exploration of the molecular metabolic mechanisms underlying the potential effects of the composite starter.

## 5. Conclusions

In this study, composite starter culture fermentation was applied to air-dried mutton processing to systematically investigate the intrinsic relationships between product quality attributes, microbial community structure, fatty acid composition, and flavor characteristics. The results demonstrated that fermentation with composite starter cultures effectively improved the overall quality of air-dried mutton, enhancing its food safety and palatability; concurrently, the fermentation reshaped the product’s microbial community structure, with *Pediococcus* and *Lactobacillus* emerging as the dominant genera, inhibiting the proliferation of spoilage bacteria and establishing a stable fermentation microecosystem. Furthermore, fermentation optimized the fatty acid composition, reduced lipid oxidation, increased the diversity of volatile flavor compounds, and elevated the concentrations of key flavor components. The study further confirmed that LAB-dominated microbiota act as key regulators of fatty acid metabolism and flavor formation in air-dried mutton, providing theoretical and technical support for the industrial production and quality improvement of traditional air-dried mutton.

## Figures and Tables

**Table 1:** Sensory evaluation criteria.

Evaluation Indicators	19–25 Points	13–18 Points	7–12 Points	1–6 Points
Color	Bright red or rose red	Brownish red	Dark red	Excessively dark, appearing dark red
Odor	Rich in characteristic flavor of fermented dried meat or dried meat	Possesses characteristic flavor of fermented dried meat or dried meat, free of off-odors	Normal meaty odor with faint fermented or dried meat flavor, moderate aroma	Poor meaty odor, no characteristic flavor, with off-odors
Tissue Structure	Neat cross-section with slight indistinct cracks	Neat cross-section with distinct cracks	Irregular cross-section with cracks	Irregular cross-section with obvious cracks
Texture	Moderate hardness	Relatively firm	Slightly loose	Loose

**Table 2:** Physicochemical quality indices for fermented air-dried meat.

Physical and Chemical Properties	ZR	FJ	*p*-Value
pH	5.85 ± 0.01 ^a^	5.40 ± 0.01 ^b^	<0.001
Aw	0.68 ± 0.01 ^a^	0.60 ± 0.01 ^b^	<0.001
Moisture Content (%)	8.34 ± 0.02 ^a^	6.01 ± 0.02 ^b^	<0.001
L*	45.62 ± 0.38 ^b^	49.77 ± 2.83 ^a^	0.012
a*	11.16 ± 0.33 ^b^	13.51 ± 0.33 ^a^	<0.001
b*	16.11 ± 0.31 ^a^	15.08 ± 0.13 ^b^	0.002
TVB-N Content	10.35 ± 0.30 ^a^	5.36 ± 0.24 ^b^	<0.001
TBARS Content	0.67 ± 0.01 ^a^	0.34 ± 0.02 ^b^	<0.001
Taste Profile	19.51 ± 0.39 ^b^	20.00 ± 0.21 ^a^	0.031
Flavor Characteristics	19.40 ± 0.16 ^b^	22.60 ± 0.19 ^a^	<0.001
Morphological Structure	18.97 ± 0.23 ^b^	20.11 ± 0.24 ^a^	<0.001
Color Attributes	18.63 ± 0.10 ^b^	21.50 ± 0.13 ^a^	<0.001

Note: All values are expressed as means ± standard deviations (SDs). Different lowercase letters within the same row indicate significant differences (*p* < 0.05) between the two groups (ZR and FJ), as determined via Duncan’s multiple range test. L*, a*, b* are instrumental color parameters representing lightness, redness, and yellowness, respectively, determined as described in the instrumental color analysis; Taste Profile, Flavor Characteristics, Morphological Structure, and Color Attributes are sensory evaluation indicators assessed in Section 2.4.3, with scoring criteria detailed in Table 1.

**Table 3:** Sequence information and diversity values of fermented air-dried meat.

Sample Information	Sequence Number	Sequence Length	ASV	Sobs	Ace	Chao	Shannon	Simpson	*p*-Value
ZR	47,561	421	263	80.33	82.28	82.50	2.25	0.19	-
FJ	52,366	429	118	22.00	22.82	22.17	1.81	0.22	<0.001 (ASV); <0.001 (Ace); <0.001 (Chao); 0.008 (Shannon); 0.321 (Simpson)

**Table 4:** Free Fatty Acid Content in Fermented and Air-Dried Meat.

Name	FJ	ZR	*p*-Value
γ-Linolenic acid (C18:3n-6)	0.56 ± 0.02 ^b^	19.76 ± 14.05 ^a^	0.021
Docosanoic acid (C22:0)	0.1 ± 0.07 ^b^	7.11 ± 5.42 ^a^	0.035
Arachidic acid (C20:0)	1.05 ± 0.53 ^a^	1 ± 0.62 ^a^	0.912
trans-9-Octadecenoic acid (C18:1)	28.11 ± 1.21 ^a^	1.66 ± 0.87 ^b^	<0.001
trans-Linoleic acid (C18:2n6t)	12.46 ± 0.99 ^a^	4.71 ± 2.07 ^b^	0.003
Heptadecanoic acid (C17:0)	0.33 ± 0.11 ^a^	0.3 ± 0.16 ^b^	0.047
Tridecanoic acid (C13:0)	1.41 ± 0.05 ^a^	0.45 ± 0.31 ^b^	0.002
Tetradecanoic acid (C14:0)	3.78 ± 0.05 ^a^	0.93 ± 0.06 ^b^	<0.001
Pentadecanoic acid (C15:0)	0.48 ± 0.05 ^b^	1.60 ± 0.16 ^a^	<0.001
cis-10-Heptadecenoic acid (C17:1)	0.96 ± 0.05 ^a^	0.94 ± 0.22 ^a^	0.876
cis-11,14-Eicosadienoic acid (C20:2)	0.2 ± 0.06 ^b^	21.21 ± 2.96 ^a^	<0.001
cis-11-Eicosenoic acid (C20:1)	0.7 ± 0.06 ^b^	8.95 ± 1.24 ^a^	<0.001
Docosadienoic acid (C22:2)	0.50 ± 0.2 ^b^	8.86 ± 1.20 ^a^	<0.001
cis-15-Tetracosenoic acid (C24:1)	0.12 ± 0.01	ND	–
cis-8,11,14-Eicosatrienoic acid (C20:3n6)	2.70 ± 0.56 ^a^	0.8 ± 0.43 ^b^	0.018
Lauric acid (C12:0)	1.11 ± 0.71 ^a^	0.20 ± 0.16 ^b^	0.042
Palmitic acid (C16:0)	22.99 ± 0.54 ^a^	1.02 ± 0.17 ^b^	<0.001
Palmitoleic acid (C16:1)	0.94 ± 0.03 ^b^	14.40 ± 8.01 ^a^	0.023
Linoleic acid (C18:2)	ND	3.20 ± 1.5	–
Heneicosanoic acid (C21:0)	ND	ND	–
Stearic acid (C18:0)	9 ± 0.66	ND	–
Oleic acid (C18:1)	0.46 ± 0.05 ^b^	1.71 ± 0.7 ^a^	0.015
Eicosapentaenoic acid (C20:5)	0.03 ± 0.01	ND	–
Capric acid (C10:0)	11.97 ± 3.01 ^a^	0.07 ± 0.01	<0.001
Tetracosanoic acid (C24:0)	ND	1.17 ± 0.15	–
SFA	52.22 ± 5.26 ^a^	13.85 ± 6.03 ^b^	<0.001
MUFA	31.29 ± 1.01 ^b^	27.66 ± 12.03 ^a^	0.038
PUFA	16.45 ± 2.61 ^b^	58.54 ± 21.93 ^a^	0.009
PUFA/SFA	0.32 ± 0.05 ^b^	4.23 ± 1.62 ^a^	0.007
n6/n3	12.34 ± 2.11 ^a^	–	–
AI	0.42 ± 0.03 ^b^	0.87 ± 0.12 ^a^	0.002
TI	0.51 ± 0.04 ^b^	1.23 ± 0.21 ^a^	0.001
h/H	2.15 ± 0.18 ^a^	0.89 ± 0.11 ^b^	<0.001

Note: All values are expressed as means ± standard deviations (SDs). Different lowercase letters within the same row indicate significant differences (*p* < 0.05) between the two groups (ZR and FJ), as determined via Duncan’s multiple-range test. Values with the same letter within the same row are not significantly different (*p* ≥ 0.05). “ND” indicates that the corresponding index or volatile compound was not detected in the sample.

**Table 5:** ROAV values and types of aromas of flavor compounds in fermented air-dried meat.

Name	Sensory Threshold	ROAV		Aroma Type
		ZR	FJ	
1-Octanol	110	0.00	0.01	Waxy aroma, Floral aroma, Fruity aroma
1-Octen-3-ol	10	0.24	0.20	Mushroom aroma, lavender aroma, hay-like aroma
2,3-Butanediol	1500	ND	1.86	Milky aroma, floral aroma, sweet aroma
2-Ethylhexanol	89	0.02	0.01	Citrus aroma, sweet aroma
1-Heptanol	330	0.00	0.00	Herbal aroma, fruity aroma
1-Hexanol	100	0.07	0.01	Fruity aroma, floral aroma, grass-like aroma
Heptanal	3	1.91	1.68	Roasted aroma, nutty aroma, fresh aroma
Decanal	2	0.23	0.23	Citrus aroma, floral aroma
Hexanal	4.5	1.68	1.22	Fruity aroma, grass-like aroma
Nonanal	0.13	100.00	100.00	Rose-like aroma, citrus aroma
Isovaleraldehyde	0.4	0.00	0.65	Fruity aroma, grass-like aroma
3-Methylhexanal	0.7	8.20	0.00	Nutty aroma
3,5-Octadien-2-one	70	0.01	0.00	Mushroom aroma, floral aroma
2,5-Octanedione	0.54	0.00	3.33	Grass-like aroma, milky aroma
Vinyl hexanoate	0.05	50.82	35.93	Green aroma, fruity aroma
Ethyl acetate	100	ND	3.21	Fruity aroma, wine aroma
Ethyl octanoate	5	ND	1.87	Fruity aroma, wine aroma, creamy aroma
Ethyl decanoate	10	ND	2.15	Fruity aroma, milky aroma, honey aroma
2,3,5,6-Tetramethylpyrazine	6000	ND	1.08	Nutty aroma, roasted aroma
3-Methyloxirane-2-carboxylic acid	20	0.00	0.01	Grass-like aroma, fruity aroma
1-Caryophyllene	0.1	1.68	4.49	Clove-like aroma, peppery aroma
Valencene	0.13	0.00	0.64	Citrus aroma

Note: “ND” indicates that the corresponding index or volatile compound was not detected in the sample.

## Data Availability

The original contributions presented in this study are included in the article. Further inquiries can be directed to the corresponding authors.

## Abbreviations

The following abbreviations are used in this manuscript:

TVB-N	Total volatile basic nitrogen
TBARS	Thiobarbituric acid reactive substances
ROAV	Relative odor activity value
Aw	Water activity
MgO	Magnesium oxide
TCA	Trichloroacetic acid
CCS	Circular consensus sequencing
SPME	Solid-phase microextraction
GC-MS	Gas chromatography–mass spectrometry
PC	Principal component
